# Selenium-binding protein 1 is down-regulated in malignant melanoma

**DOI:** 10.18632/oncotarget.23853

**Published:** 2018-01-02

**Authors:** Mandy Schott, Miriam M. de Jel, Julia C. Engelmann, Philipp Renner, Edward K. Geissler, Anja K. Bosserhoff, Silke Kuphal

**Affiliations:** ^1^ University of Erlangen, Institute of Biochemistry, Biochemistry and Molecular Medicine, Erlangen, Germany; ^2^ University of Regensburg, Institute of Functional Genomics, Statistical Bioinformatics, Regensburg, Germany; ^3^ Department of Surgery, University Medical Center Regensburg, Regensburg, Germany

**Keywords:** malignant melanoma, selenium-binding protein 1 (SELENBP1), glutathione peroxidase 1 (GPX1), Grm1 mouse model

## Abstract

Selenium-binding protein 1 (SELENBP1) expression is reduced in various epithelial cancer entities compared to corresponding normal tissue and has already been described as a tumor suppressor involved in the regulation of cell proliferation, senescence, migration and apoptosis. We identified SELENBP1 to be down-regulated in cutaneous melanoma, a malignant cancer of pigment-producing melanocytes in the skin, which leads to the assumption that SELENBP1 also functions as tumor suppressor in the skin, as shown by others e.g. for prostate or lung carcinoma.

However, *in vitro* analyses indicate that SELENBP1 re-expression in human melanoma cell lines has no impact on cell proliferation, migration or tube formation of the tumor cells themselves when compared to control-transfected cells. Interestingly, supernatant taken from melanoma cell lines transfected with a SELENBP1 re-expression plasmid led to suppression of vessel formation of HMEC cells. Furthermore, SELENBP1 re-expression alters the sensitivity of melanoma cells for Vemurafenib treatment.

The data also hint to a functional interaction of SELENBP1 with GPX1 (Glutathione peroxidase 1). Low SELENBP1 mRNA levels correlate inversely with GPX1 expression in melanoma. The re-expression of SELENBP1 combined with down-regulation of GPX1 expression led to reduction of the proliferation of melanoma cells. In summary, SELENBP1 influences the tumor microenvironment and SELENBP1 action is functionally influenced by GPX1.

## INTRODUCTION

Malignant melanoma is the most aggressive form of skin cancer, and its incidence is rising at alarming rates [1]. To study melanoma development and progression *in vivo* the transgenic mouse strain Tg(*Grm1*) was generated [2]. Tg(*Grm1*) mice spontaneously develop pigmented lesions within a short time of latency and with 100% penetrance due to the melanocyte-specific expression of a *metabotropic glutamate receptor 1* (*Grm1*) transgene. As the *Grm1* transgene is placed under control of the melanocyte specific *Dct* promoter, *Grm1* is specifically overexpressed in cells of melanocytic origin leading to both cutaneous and uveal melanoma [2, 3]. Interestingly, GRM1 up-regulation is also present in human melanoma cell lines and tissues [2, 4, 5]. Since transcriptome sequencing did not reveal melanoma-associated mutations or single nucleotide variations in Tg(*Grm1*) mice [6], we assume, besides *Grm1* overexpression, epigenetic events or changes in gene expression are important for driving melanoma development and progression in this mouse model.

Selenium is a micronutrient for a number of physiological biological processes in the human body. Selenium supplementation at nutritional dosage (nM range) has been extensively studied for its preventive effects against various cancers [7–9], implicating that selenium-containing proteins are likely to play crucial roles in selenium-mediated cancer prevention. Selenium-binding protein 1 (*SELENBP1, SBP1, hsP56*), a member of the selenoprotein family, has been shown to mediate the intracellular transport of selenium [10]. SELENBP1 is expressed in a wide range of normal human tissues, but is suppressed in diverse types of epithelial cancers such as prostate, stomach, colon, lung, thyroid and ovary [11–14]. SELENBP1 down-regulation is associated with tumor progression as well as poor clinical outcome [15–18]. Moreover, several studies show that SELENBP1 is involved in the regulation of cellular processes including proliferation, migration, senescence and apoptosis [16, 18–20].

Glutathione peroxidase 1 (GPX1) is also an important selenium-containing protein which is ubiquitously expressed. GPX1 is an antioxidant-enzyme that scavenges organic hydroperoxides using reducing equivalents from glutathione and protects cells from reactive oxygen species (ROS) [21–23]. Among the 25 human selenocysteine-containing proteins, there is considerable evidence that the cytosolic form of GPX1 is associated with cancer risk. Given the cumulative data indicating possible roles of both SELENBP1 and GPX1 in cancer development and/or outcome, the interaction of these two selenium-associated proteins was investigated in several model systems [24].

In the present study we demonstrate that SELENBP1 is not only down-regulated in malignant melanoma samples of a murine Tg(*Grm1*) model for spontaneous melanoma but also in human melanoma cell lines and tissues, primarily arguing that SELENBP1 may be an important tumor suppressor in malignant melanoma. Interestingly, no direct influence of SELENBP1 re-expression on melanoma cells themselves was observed. However, SELENBP1 re-expression changes tube formation capacity of HMEC cells thus having an effect on the tumor microenvironment in melanoma.

## RESULTS

### SELENBP1 is suppressed in murine melanoma tissues of the Tg(*Grm1*) mouse model

**Figure 1:**
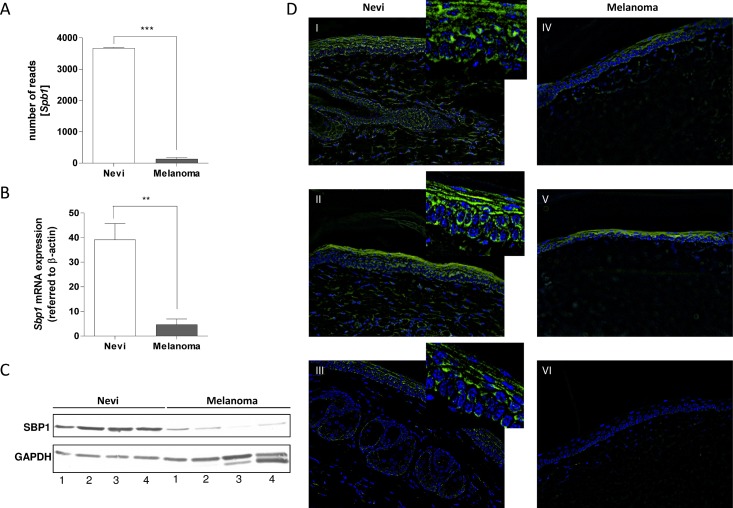
SELENBP1 expression in murine Tg(*Grm1*) melanoma tissue (**A**) *SELENBP1* (*Sbp1*) expression in murine Tg(*Grm1*) melanoma (*n* = 2) compared to nevi (*n* = 2) tissue via RNA-sequencing analysis. (**B**) Quantitative real-time PCR analysis to calculate mRNA expression in murine nevi (*n* = 5) and murine melanoma samples (*n* = 5) (^**^p-value: 0.0011). (**C**) Western blot analysis to detect SelenBP1 on protein level in murine melanoma tissue samples (*n* = 4) compared to nevi samples (*n* = 4). GAPDH was used as a loading control. (**D**) Immunofluorescence analysis of SelenBP1 (green) in murine nevi tissue from the tail (I, II, III) and primary melanomas (IV, V, VI). DAPI was used to visualize the localization of nuclei. All images showed unspecific green staining of the epidermal keratin layer.

To analyze differences in gene expression profiles between nevi and melanoma samples, a RNA-sequencing analysis was performed on two nevi and two melanoma samples from Tg(*Grm1*) mice. The sequence data is publicly available from NCBI, BioProject PRJNA237546. In this analysis *SelenBP1* was found to be one of the strongest down-regulated gene of a total of 1,085 down-regulated genes in Tg(*Grm1*) melanoma samples compared with nevi tissue. Whereas nevi samples reach a number of 3,674 reads on average, melanoma samples display a mean number of only 132 reads for the *SelenBP1* gene (Figure 1A). The lack of SelenBP1 data regarding its role in melanoma prompted us to analyze its relevance in this kind of cancer. Studies with additional tissue samples confirmed *SelenBP1* down-regulation in murine melanoma samples both on mRNA (Figure 1B) and protein level (Figure 1C). The murine nevi and tumor samples showed strong pigmentation; therefore, it was necessary to perform immunofluorescence instead of immunohistochemistry. Immunofluorescence analyses with murine nevi samples of the tail indicate the specificity of SelenBP1 staining (green) in pigmented melanocytes (Figure 1D), whereas melanomas are only positively stained for DAPI (localization of the nuclei). Hence, this *in vivo* result reveals down-regulation of SelenBP1 in murine melanoma samples.

### SELENBP1 is down-regulated in human melanoma cell lines and tissues

**Figure 2:**
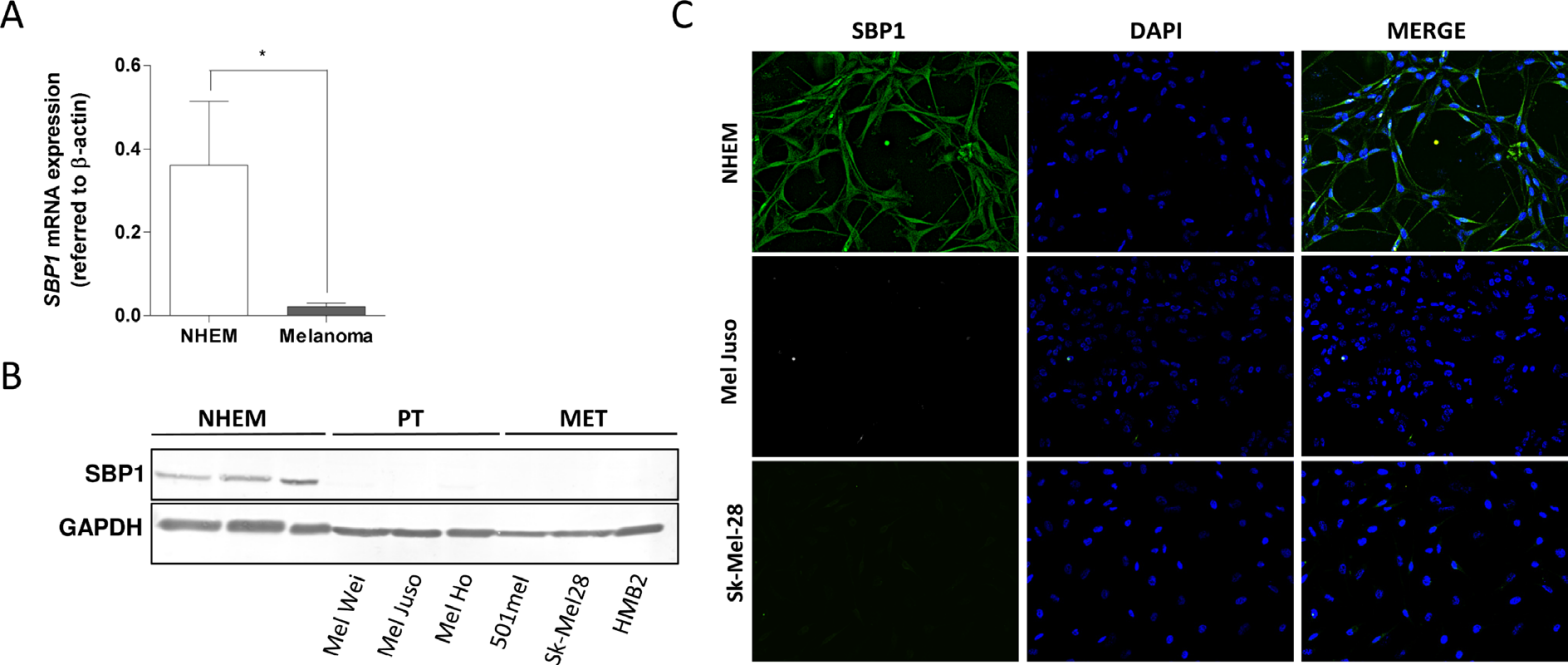
SELENBP1 in human melanoma cell lines (**A**) *SELENBP1* mRNA expression in melanoma cell lines (*n* = 10) and normal human epidermal melanocytes (NHEM) (*n* = 8). *SELENBP1* expression levels were normalized to β-*actin* (^*^p-value: 0.0228). (**B**) Western blot analysis for detecting SELENBP1 protein in melanoma cell lines and NHEM. GAPDH served as a loading control. (**C**) Immunofluorescence staining for SELENBP1 protein using representative melanoma cell lines Mel Juso and Sk-Mel-28 and NHEM. DAPI was used for nuclear detection.

To investigate whether data obtained from the murine melanoma mouse model are relevant to the human system, qRT-PCR analyses were performed with normal human epidermal melanocytes (NHEM) and human melanoma cell lines. Compared to NHEM, melanoma cells display a significant decrease in *SELENBP1* mRNA expression (Figure 2A). SELENBP1 suppression in melanoma cells (PT, primary tumor; MET, metastasis) was confirmed on protein level showing reduced SELENBP1 protein amounts in western blot analysis compared to healthy NHEM (Figure 2B), and in immunofluorescence staining of two representative cell lines (Figure 2C).

Silencing of the *SELENBP1* gene due to hypermethylation and chromatin remodeling appears to be frequently involved in tumorigenesis of colorectal cancer [25]. To further investigate the roles of CpG island methylation and histone deacetylation in transcriptional silencing of *SELENBP1* in melanoma, we induced DNA demethylation and inhibited histone deacetylase. The cell lines were incubated with 5 μM 5-Aza-deoxycytidine for 72 h, followed by treatment with 300 nM TSA for 4 h. The treatment of the cells did not lead to an induction of *SELENBP1* expression (Supplementary Figure 1A). These results suggest that epigenetic silencing by hypermethylation of the *SELENBP1* promoter is not involved in its regulation in melanoma. Speculating that a *SELENBP1* gene mutation could be responsible for the low expression levels, we studied the COSMIC (Catalogue of Somatic Mutations in Cancer)-home page [26, 27]. Only 13 of 1,009 analyzed malignant melanoma samples in total harbor a *SELENBP*1 mutation (data not shown). The result was supported by the finding that RNA-sequencing analysis of the Tg(*Grm1*) mouse model uncovered also no melanoma-associated mutations or single nucleotide variations [6].

Next, we speculated that *SELENBP1* can be regulated by hypoxia, a condition which is cumulative and endogenously found in melanomas. A previous publication of our own group revealed constitutively induction of HIF-1α expression in melanoma cells [28]. Therefore, we investigated the hypoxic effects on *SELENBP1* expression using Desferrioxamine (DFX) and 2, 2′-dipyridyl (DP) as iron chelators and inhibitors of prolyl hydroxylases (PHDs). Both chemical compounds mimic hypoxic effects and increase the *SELENBP1* amount on mRNA level as exemplarily shown for the melanoma cell lines Sk-Mel-28 and Mel Juso (Supplementary Figure 1B). Hence, hypoxic effects induce *SELENBP1* expression.

### SELENBP1 is down-regulated in human melanoma *in vivo*

**Figure 3:**
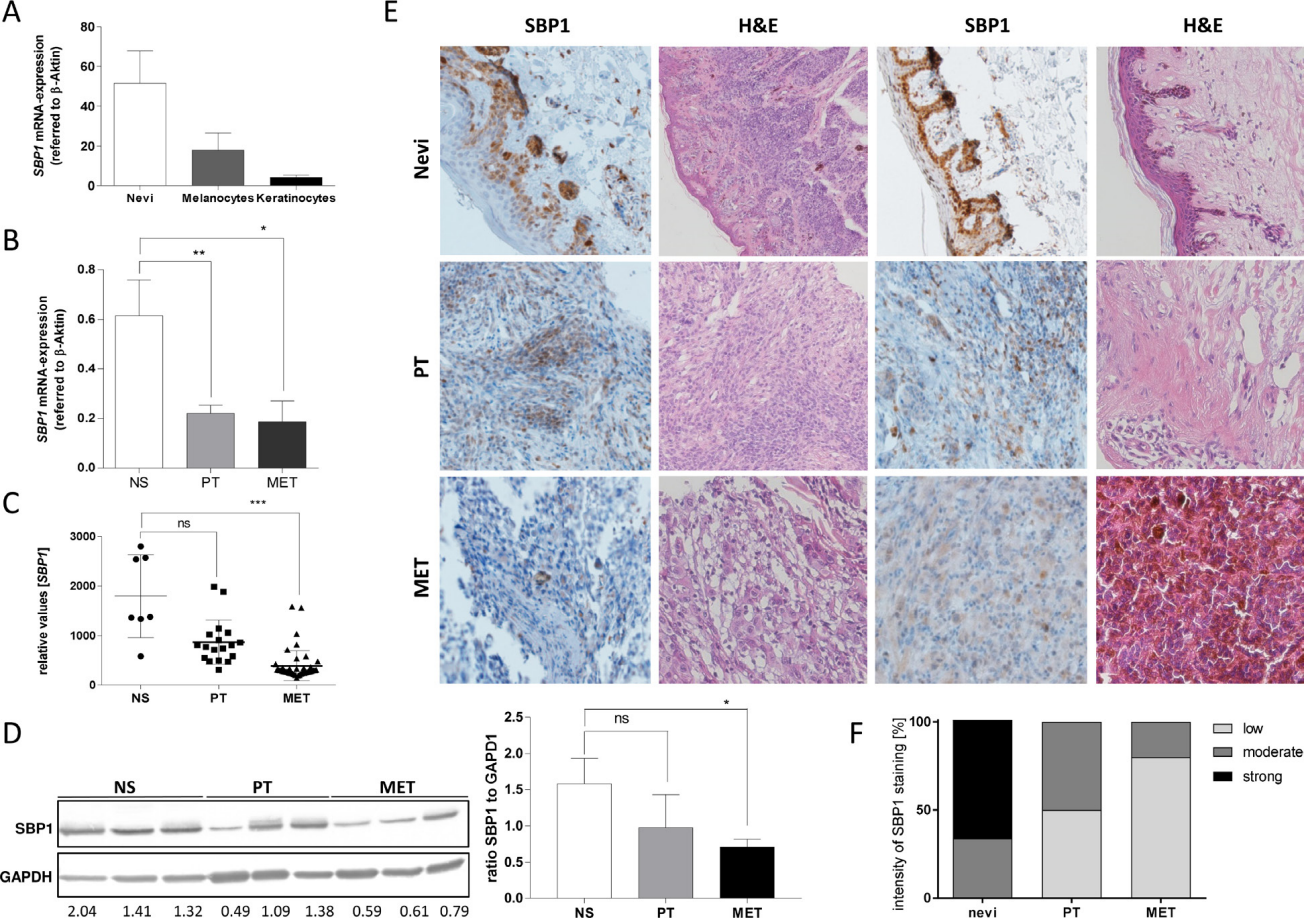
SELENBP1 *in vivo* in human melanoma (patient) (**A**) Expression analysis of *SELENBP1* in nevi, melanocytes and keratinocytes. (**B**) Tissue of normal skin (*n* = 4), primary melanoma (*n* = 4) and melanoma metastasis (*n* = 4) were analyzed for *SELENBP1* expression on mRNA level. (**C**) Geoprofile data sets (GDS1375) showed *SELENPB1* mRNA expression in normal skin (NS) and in primary tumor (PT) and metastasis (MET) of melanoma patient. (**D**) SELENBP1 protein level was analyzed by Western blot analysis. Values below the blot indicate the ratio between SELENBP1 and GAPDH for each sample and were calculated using ImageJ. (**E**) Immunohistochemical analysis showed SELENBP1 staining in primary melanoma biopsies (*n* = 5) and melanoma metastases (*n* = 5) compared to nevi tissue (*n* = 6). Representative microscopy images were shown for each tissue, as well as H&E staining. (**F**) Statistical analysis of SELENBP1 immunohistochemistry. Evaluation was performed by a classification into three categories: low, moderate and high SELENBP1 protein expression.

To analyze *SELENBP1* expression in human *in vivo* samples in general, we first performed qRT-PCR with mRNA samples from melanoma patients from isolated nevi tissue, isolated primary melanocytes and keratinocytes (Figure 3A). Nevi and melanocytes showed high expression levels of *SELENBP1*, while keratinocytes harbor lower levels of *SELENBP1* mRNA. In addition, *SELENBP1* mRNA expression was analyzed in human tissue samples from melanoma patients, displaying a decrease in *SELENBP1* mRNA compared to normal skin (NS) (Figure 3B). Evaluating geoprofile data (GDS1375) from melanoma patients confirmed the previous results that normal skin samples (NS) express high amounts of *SELENBP1* mRNA compared to primary tumor and metastasis samples (Figure 3C). Analyzing protein data by western blot (Figure 3D) confirmed the results evaluated on mRNA level. SELENBP1 is stronger expressed in normal skin (NS) compared to melanoma metastases (MM). We furthermore aimed to analyze SELENBP1 by immunohistochemistry experiments on a tissue microarray (TMA) (Figure 3E). Here, ten normal, respectively nevi skin samples, ten primary tumor samples and ten melanoma metastases were spotted on the TMA. Exemplarily, shown are two stainings of each type. To discriminate between a possible melanin deposit and the brown HRP-detection color we presented also the corresponding H&E staining of the samples (Figure 3E). The images of specific SELENBP1 (SBP1) staining illustrate again the elevated SELENBP1 expression in normal skin and nevi and the decrease of SELENBP1 in primary melanoma and metastatic melanoma patient tissue. In the corresponding graphical analysis (Figure 3F) we quantified the SELENBP1 staining of the whole TMA. Strong staining was detectable in 66 % of nevi samples by contrast 50 to 80 % of melanoma cases showed SELENBP1 protein staining with low intensity.

### SELENBP1 re-expression in human melanoma cells

**Figure 4:**
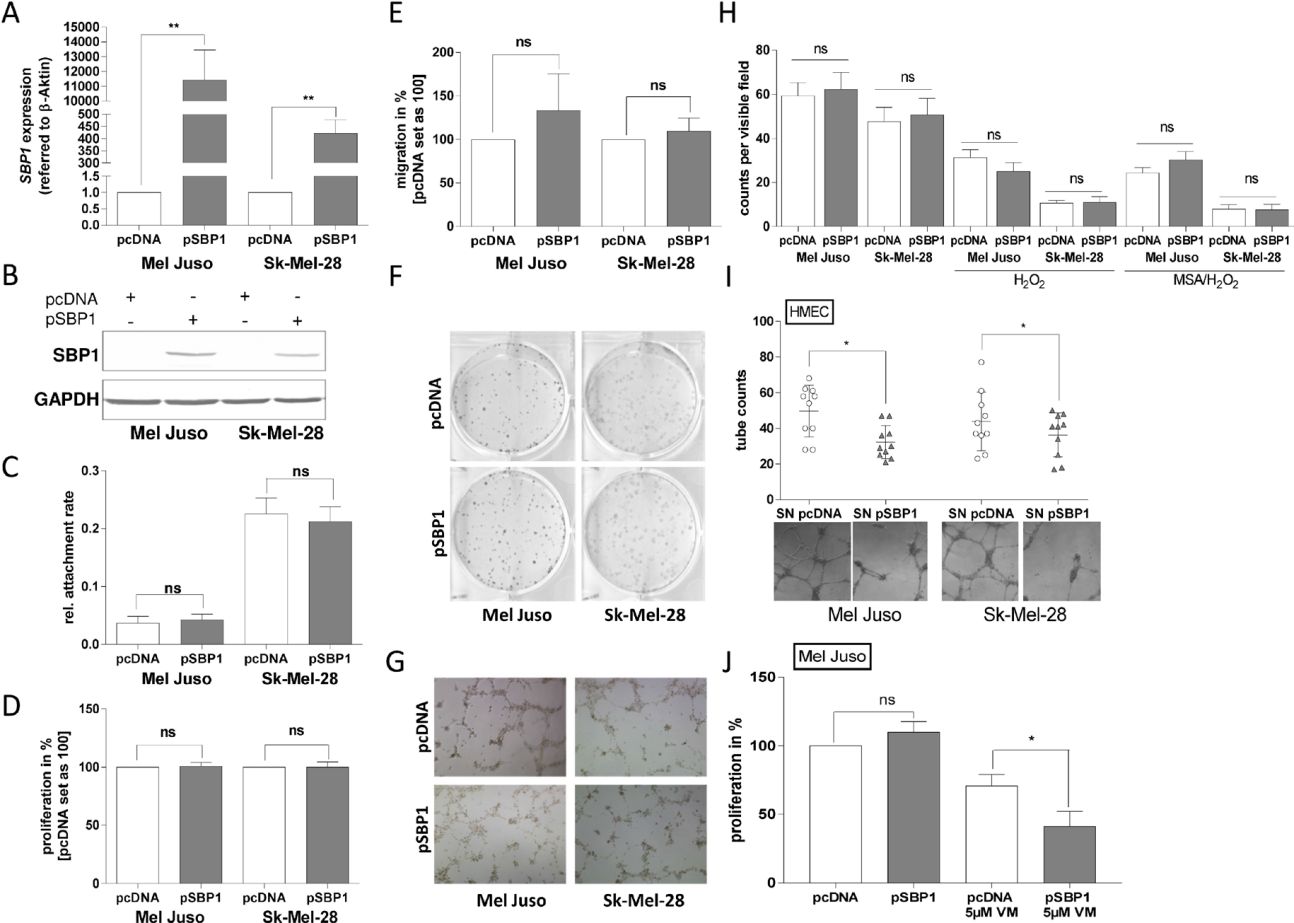
SELENBP1 re-expression in human melanoma cells and cellular mechanisms (**A**) Quantitative real-time PCR analysis and (**B**) Western blot analysis confirmed SELENBP1 (pSBP1) re-expression in Mel Juso and Sk-Mel-28 melanoma cell lines, compared to pcDNA control-transfected cells 24 h after transfection (^**^p < 0.01). (**C**–**E**) RTCA experiments to analyze alterations in cell attachment, proliferation and migration (ns: not significant). (**F**) Clonogenic assays for analyzing the impact of SELENBP1 re-expression on self-renewing capacity. (**G**) Matrigel-based tube formation assays displayed the development of vascular channels after transfection with pSBP1 vector. (**H**) MSA and H_2_O_2_ treatment together with pcDNA or SELENBP1 (pSBP1) over-expression, respectively, was analyzed by RTCA (ns: not significant). (**I**) Supernatant (SN) of melanoma cell lines re-expressing SELENBP1 (pSBP1) and control transfected cells (pcDBNA) was used for cell culture of human dermal microvascular endothelial cells (HMECs). Matrigel-based tube formation assays displayed the vessel formation. (**J**) RTCA proliferation assay for the melanoma cell line Mel Juso treated with Vemurafenib (5 μM) and transfected with the SELENBP1 expression construct, respectively.

To define the role of SELENBP1 in malignant melanoma, the primary melanoma cell line Mel Juso and the metastatic cell line Sk-Mel-28 were transiently transfected with a SELENBP1 expression plasmid (pSBP1). After 24 h, SELENBP1 re-expression was confirmed on both mRNA (Figure 4A) and protein level (Figure 4B). Using the RTCA system neither alterations in cell attachment (Figure 4C), nor changes concerning proliferation (Figure 4D) and migration (Figure 4E), were evident from SELENBP1 re-expression (pSBP1) when compared with pcDNA control-transfected cells. In addition, SELENBP1 re-expression seems to have no impact on self-renewing capacity, as clonogenic forming ability of single cells was not affected (Figure 4F). Moreover, the potential to form vascular-like structures was not influenced by SELENBP1 re-expression, according to results from matrigel-based tube formation assays (Figure 4G). Also in the presence of methylselenic acid (MSA-a selenium metabolite) and H_2_O_2_ (a ROS inducer), clonogenic forming ability of melanoma cells was not altered by transfection with pSBP1 (Figure 4H). In summary, SELENBP1 over-expression had no significant influence on the cancerous behavior of the melanoma cell lines themselves.

The literature suggests the involvement of SELENBP1 in regulating extracellular glutathione (GSH) [29]. We therefore speculated that SELENBP1 overexpression in melanoma cell lines influences the composition of extracellular factors in the medium of the cell lines in culture. Using the supernatant (SN) of pSBP1 transfected cells and transferring it to human dermal microvascular endothelial cells (HMEC) strongly influenced the formation of vascular structures. HMEC cells developed tubes to a lesser and thinner extend using supernatant from SELENBP1 expressing melanoma cells compared to pcDNA control transfected cells (Figure 4I). The expression of specific factors involved in epithelial to mesenchymal transition (EMT) was not affected by the supernatant (SN) of pSBP1 expressing cells, treating keratinocytes (HaCaT), fibroblasts (F V) or HMEC cell lines and analyzing the expression level of Vimentin, CDH-1 and CDH-2 on mRNA level (Supplementary Figure 1C–1E). In summary, SELENBP1 has influence on so far unknown extracellular factors of melanoma cells and influences endothelial cells, like HMECs in a paracrine manner. Although SELENBP1 re-expression has no direct influence on melanoma cells themselves (Figure 4A–4H), we treated SELENBP1 re-expressing melanoma cells with Vemurafenib a kinase inhibitor used in the treatment of patients with unresectable or metastatic melanoma to mimic a “stress” situation of the cells. The combination led to sensitization of Mel Juso melanoma cells for Vemurafenib treatment (Figure 4J).

### Correlation between SELENBP1 and GPX1

**Figure 5:**
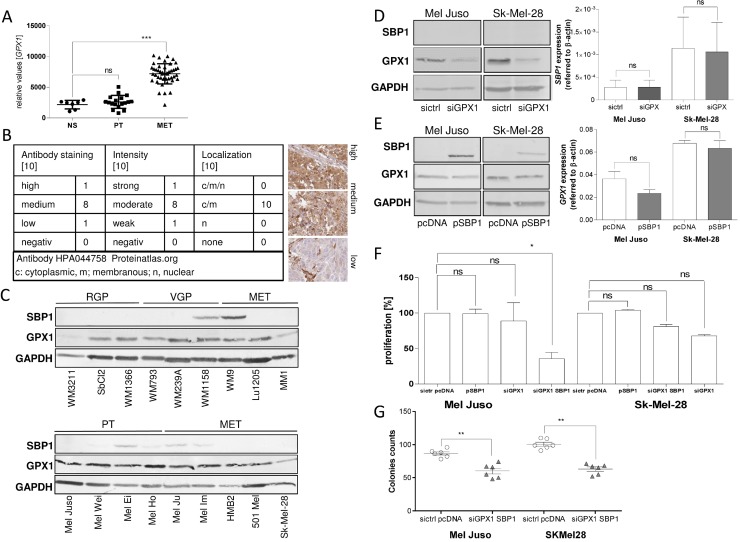
Connection between SELENBP1 and GPX1 (**A**) Geoprofile data sets (GDS1375) show *GPX1* mRNA expression in normal skin (NS), primary tumor (PT) and metastasis (MET) of melanoma patients (^***^p < 0.001; ns, not significant). (**B**) Immunohistochemical analysis showing GPX1 staining in human melanoma samples (*n* = 10, data bank of proteinatlas.org). Representative microscopy images are shown. Evaluation was performed by classification into three categories: low, medium and high GPX1 protein expression. (**C**) Western blot analysis of SELENBP1 and GPX1 protein in 18 different melanoma cell lines of different tumor stages (RGP: radial growth phase; VGP: vertical growth phase; PT: primary tumor; MET: metastasis). GAPDH was used as a loading control. (**D**) Treatment of cells with siRNA against GPX1 and confirmation of successful knock-down by using anti-GPX1 antibody. Analysis of the influence of silenced GPX1 on SELENBP1 expression using an anti-SELENBP1 antibody and analyzing the SELENBP1 expression on mRNA level. (**E**) Treatment of cells with a re-expression vector (pSBP1) for SELENBP1 and confirmation of successful re-expression by using anti-SELENBP1 antibody. Analysis of the influence of re-expressed SELENBP1 on GPX1 expression using an anti-GPX1 antibody and analyzing the GPX1 expression on mRNA level. (**F**) RTCA proliferation assay after SELENBP1 re-expression of GPX1 silencing alone and in combination. (**G**) Clonogenic assays for analyzing the impact of SELENBP1 re-expression and GPX1 knock-down on self-renewing capacity.

Geoprofile data revealed significantly elevated levels of *GPX1* expression on an mRNA level in malignant melanoma (MET), compared to normal skin (Figure 5A); protein expression data confirmed this finding. Immunohistochemistry showed medium to high expression of GPX1 mainly in the cytoplasm and nucleus (proteinatlas.org) (Figure 5B).

We investigated the expression status of GPX1, another selenium-associated protein shown to be involved in SELENBP1 signaling pathways. Western blot analysis of radial growth phase (RGP), vertical growth phase (VGP), primary tumors which were not classified (PT) and melanoma metastasis (MET) cell lines showed high GPX1 expression in 16 of 18 tested cell lines. 6 of 18 tested melanoma cell lines showed moderate SELENBP1 expression. Both molecules are not expressed in BRAF V600E dependency (Figure 5C). The two melanoma cell lines WM1158 and WM9 showed highest SELENBP1 expression without inverse correlation to GPX1 protein amount.

As it was published in literature that SELENBP1 and GPX1 are influencing each other, directly [24], we transfected melanoma cell lines with siRNA against GPX1 or an over-expression construct for SELENBP1, respectively. Analyzing the expression status on protein and mRNA level, gave no hint either for a direct influence of GPX1 on SELENBP1 expression (Figure 5D) or of SELENBP1 on GPX1 expression (Figure 5E).

Interestingly, we combined re-expression of SELENBP1 and silencing of GPX1 and achieved a significant reduction of proliferation up to 70% in the cell line Mel Juso (Figure 5F). Sk-Mel-28 only showed a tendency for down-regulated proliferation after a combined manipulation of SELENBP1 and GPX1 expression (Figure 5F). Analyzing the influence of SELENBP1 re-expression and simultaneous GPX1 down-regulation in clonogenic assays led to the result that a combination of both molecules reduces the amount of colonies (Figure 5G).

## DISCUSSION

SELENBP1 is suppressed in several human cancers including cancers of the prostate, lung, breast and ovary [11–14]. Indeed, SELENBP1 is described as a tumor suppressor in these tumor types, since decreased levels are associated with enhanced cell proliferation and migration, as well as inhibited apoptosis. Moreover, SELENBP1 re-expression results in diminished cancer cell proliferation and migration, and an induction of apoptosis in colorectal and breast cancer [13, 18, 19].

Transgenic Tg(*Grm1*) mice serve as a murine model system for spontaneous melanoma development and provide some benefits compared with other melanoma models, avoiding the complication of using and having the feature of metastasis to distant organs including lung and liver [3]. Furthermore, Tg(*Grm1*) mice offer the possibility to compare nevi and melanoma tissue from the same genetic background with respect to changes in the gene expression profile. This mouse model provided the first clues of an important role of SELENBP1 in melanoma and prompted us to further examine this gene in humans. The present study is the first to demonstrate that SELENBP1 is downregulated in human malignant melanoma compared to normal tissue and normal human melanocytes (NHEM). This discovery raises the hypothesis that SELENBP1 functions as tumor suppressor in cutaneous melanoma.

The reason for low or lost, respectively, SELENBP1 expression remains unknown. We excluded CpG island methylation and loss of function mutations for melanoma and speculate that transcriptional or microRNA dependent suppression is a reason for low SELENBP1 levels. As example, SELENBP1 has been identified as a target of the oxygen-responsive HIF-1 α transcription factor [30].

To characterize the consequences of SELENBP1 suppression in malignant melanoma several functional assays were performed using a human SELENBP1 expression plasmid (pSBP1). However, these *in vitro* analyses gave no evidence that SELENBP1 has a functional impact on melanoma cells themselves in terms of attachment, proliferation, migration, self-renewing capacity and tube formation. As reported by others, an inhibitory effect of SELENBP1 re-expression on proliferation in cell culture may require the MSA (a selenium metabolite) supplementation to ensure sufficient selenium in the assays [18]. As example, breast cancer cells without MSA treatment did not display altered proliferation potential upon transfection with a SELENBP1 plasmid [11]. Therefore, we incubated melanoma cells also with MSA and again detected no influence by combination of SELENPB1 re-expression and simultaneous MSA treatment. Additional studies report that altered proliferation in SELENBP1 dependency occurs only after treatment with hydrogen peroxide [20, 23]; H_2_O_2_ is a reactive oxygen species (ROS) leading to enhanced oxidative stress in cells. Enhanced ROS level can induce DNA damage of tumor cells and lead to cell death, requiring cancer cells to adopt protective mechanisms. These hints from the literature prompted us to test MSA and H_2_O_2_ treatment in human malignant melanoma cells. Neither MSA nor H_2_O_2_ treatment altered the effects of SELENBP1 transfection in malignant melanoma cells in our experiments. To proof, whether SELENBP1 exerts influences on Vemurafenib treatment of melanoma cells we incubated cells with Vemurafenib and re-expressed SELENBP1, which sensitized melanoma cells for the therapy.

Loss of SELENBP1 resulted in the high-affinity uptake of selenite through elevating the levels of extracellular GSH. Reduction of SBP1 accelerated uptake of extracellular selenite and reduction of SBP1 enhanced selenite-mediated cytotoxic effects through elevating extracellular GSH levels [29] with possible consequences also of the vessel formation of endothelial cells in the environment of cancer cells. We therefore speculated that SELENBP1 overexpression in melanoma cell lines influences the composition of extracellular factors thus influencing the tumor microenvironment. Interestingly, supernatant taken from melanoma cell lines transfected with a SELENBP1 re-expression plasmid led to suppression of vessel formation of HMEC cells.

Glutathione peroxidase (GPX1), an intracellular antioxidant enzyme, is a protector against ROS effects [31]. Interestingly, elevated GPX1 activity causes an increase in cell proliferation [21]. In previous studies, increasing the levels of SELENBP1 reduced GPX1 enzyme activity [20, 23, 24] and reducing SELENBP1 levels increased GPX1 enzyme activity. In summary, it was speculated that SELENBP1 inhibits GPX1 activity and thus may function as a tumor suppressor only by regulating GPX1. Our results did not confirm these results for melanoma. Manipulating GPX1 expression did not influence SELENBP1 mRNA and protein amount and vice versa. The genes are not regulating each other and loss of SELENBP1 and over-expression of GPX1 eventually are independent events during melanoma progression. We speculate that both molecules still compensate the function from each other.

However, simultaneous SELENBP1 over-expression plus GPX1 knockdown reduces melanoma cell proliferation significantly. These results support that SELENBP1 could be a tumor suppressor lost early in development in melanoma and it was functionally compensated by GPX1 in later melanoma progression.

In summary, our results show that data obtained from the Tg(*Grm1*) mouse model are relevant to the human system and may provide insights into molecular modifications leading to melanoma development and progression. Therefore, this innovative model system is a useful tool for unraveling new genes involved in melanomagenesis. Furthermore, SELENBP1 re-expression leads to changes in proliferation of melanoma cells when incubating it together with Vemurafenib. Additionally, we revealed that SELENBP1 changes microenvironmental factors and has paracrine influence on surrounding cell types of melanoma cells, as shown for human dermal microvascular endothelial cells (HMEC).

## MATERIALS AND METHODS

### Transgenic mice

The transgenic Tg(*Grm1*) mice were established at the Department of Chemical Biology, Rutgers University, Piscataway, USA [2] and kindly provided by Prof. Suzie Chen and Prof. Jürgen Becker. Mice were kept under standard conditions at 21°C (± 1°C) with 55% (± 10%) relative humidity and 12 h light/dark intervals. Animals were fed with standard chow (Ssniff, Soest, Germany) and with drinking water *ad libitum* [3]. Animal care and experimental procedures were carried out in accordance with the guidelines of the German law governing animal care. Experiments were approved by the Ethics Committee for Animal Research of the Bavarian government. For all analyses, we used homozygous transgenic animals bred in our laboratory.

### Cell culture and tissue samples

Human melanoma cell lines Mel Ei, Mel Wei, Mel Juso, Mel Ho (derived from primary cutaneous melanoma), Mel Ju, Mel Im, 501 Mel, Sk-Mel-28 and Hmb2 (derived from metastases of malignant melanoma) were cultured in Dulbecco's modified Eagle's medium (DMEM) supplemented with penicillin (400 U/ml), streptomycin (50 μg/ml) and 10% fetal calf serum (all from Sigma-Aldrich, München, Germany). Normal human epidermal melanocytes (NHEM) were cultivated in melanocyte growth medium M2 (PromoCell, Heidelberg, Germany). HMEC cells were cultivated in Medium 200 (Gibco, Thermo Fisher Scientific, Rockford, USA) supplemented with 50× LVES (Gibco) and penicillin (400 U/ml), streptomycin (50 μg/ml). HaCaT and FV cells were cultivated in DMEM supplemented with penicillin (400 U/ml), streptomycin (50 μg/ml) and 10% fetal calf serum. All cell lines were incubated at 37°C in a humidified atmosphere containing 8% CO_2_.

H_2_O_2_ treatment was performed at a concentration of 25 μM. Methylseleninic acid (MSA) was dissolved and added to the cell cultures as described by Liu *et al.* [32]. Vemurafenib was purchased by Absource Diagnostics (Munich, Germany). Human tissue samples of snap-frozen normal skin (TB 20, TB30, TB33, TB34), nevi, primary melanoma tumors (TB97, TB148, TB199) and melanoma metastases (Met124, Met202, Met203) with clear-cut pathological classification were obtained from our tissue collection (Institute of Pathology, University of Regensburg, Germany). Sampling and handling of patient material were carried out in accordance with the ethical principles of the Declaration of Helsinki.

### Transfection experiments

Cells were plated 2 × 10^5^ cells/well into 6-well plates and transfected with 0.5 μg plasmid DNA using the lipofectamine plus method (Invitrogen, Darmstadt, Germany), according to the manufacturer's instructions. The human SELENBP1 expression construct (pSBP1) was provided by the research group of Prof. W. Yang [19]. SiRNA against GPX1 was purchased from Qiagen (Hilden, Germany) and used in a concentration of 20 μM (stock).

### Treatment of cell with 5-Aza-2′-deoxycytidine

The melanoma cell lines were seeded at a low density of 750,000 cells in a T75 flask, 24 h before treatment. The next day cells were treated with 5 μM 5-Aza-2′-deoxycytidine (Sigma-Aldrich; dissolved in 50% acetic acid, diluted in DMEM/10% FCS) for 72 h. Control cells were incubated with the same volume of acetic acid diluted to a 50% solution with PBS also for the time period of 72 h. Following this incubation, 300 nM Trichostatin A (TSA, Sigma Aldrich) was added to fresh media for 4 h. The control cells were incubated with the same volume of PBS diluted in fresh media.

### Hypoxia

Membrane permeable Desferrioxamine (DFX) and 2, 2′-dipyridyl (DP) (purchased from SigmaAldrich) as iron chelators and inhibitors of Prolylhydroxylases were used in a concentration of 250 μM for DFX and 50 μM for DP diluted in DMEM.

### Measurement of migration, attachment, and proliferation

The xCELLigence System (Roche, Mannheim, Germany) is based on measurement of electrical impedance and permits real-time analysis of migration, attachment, and proliferation. CIM plates (migration) and E-plates (attachment and proliferation) were used and basic protocols recommended by the manufacturer were followed. The bottom chambers contained culture supernatant from human fibroblasts as chemo-attractant. Upper chambers contained serum-free DMEM. After recording background impedance, cells suspended in serum-free DMEM were added to the upper chambers (4 × 10^3^/well for migration; 2 × 10^2^/well for proliferation/attachment). Thereafter, impedance can be measured continuously over 72 h or longer. Impedance is represented by the relative and dimensionless parameter named cell index (CI). CI values = Zi-Z0/15 [Ohm]; where Z0 = impedance at the start of the experiment, and Zi = impedance at individual time points during the experiment. The normalized cell index (NCI) was calculated as the cell index CI_ti_ at a given time point (ti) divided by the cell index CI_nml_time_ at the normalization time point (nml_time). The slope is used to describe the steepness of a curve within a given time window (in our case 1.5 h (attachment) 4 h (migration, and 100 h (proliferation)).

### Clonogenic assay (stem cell behavior)

We used the clonogenic assay as the method of choice to test the survival rate based on the ability of a single cell to grow into a colony. The assay essentially tests every cell in the population for its ability to undergo “unlimited” division. The *in vitro* assay performed as described by Franken *et al.* [33]. 50 or 100 cells, respectively, were sowed in a 6-well chamber, cultivated for approximately 10 days to a colony size of ~50 cells. The colonies were counted by microscopy.

### Tube formation (vasculogenic mimicry)

Growth factor reduced Matrigel (BD Biosciences, Heidelberg, Germany) was added to eight-chamber polystyrene vessel tissue culture-treated glass slides (BD Bioscience) and allowed to gelatinize for 20 min at 37°C. To assay vasculogenic mimicry, 7 × 10^4^ melanoma cells or HMEC cells, respectively were seeded onto Matrigel-coated culture slides. Tube formation was assessed by phase contrast microscopy after 16 h and recorded with a digital camera.

### RNA isolation, reverse transcription and quantitative RT-PCR

Total RNA was isolated using the E.Z.N.A. MicroElute Total RNA Kit (Omega Bio-Tek, VWR Darmstadt, Germany) according to the manufacturer's instructions. For RNA isolation of tissue samples ceramic beads and the Precellys homogenisator (Peqlab Biotechnologies GmbH, Erlangen, Germany) were used for mechanical fragmentation. RNA concentration was measured with a NanoDrop spectrophotometer (Peqlab Biotechnology GmbH) and cDNA was generated by reverse transcription using the Super Script II Reverse Transcriptase Kit (Life Technologies, Carlsbad, USA), with each reaction containing 500 ng of total RNA. Analysis of mRNA expression was performed using quantitative Real-Time PCR on the LightCycler 480 system (Roche, Mannheim, Germany). A volume of 1 μl cDNA template, 0.5 μl of forward and reverse primers (each 20 μM) and 10 μl of SYBR Green I (Roche, Mannheim, Germany) were combined to a total volume of 20 μl. The following primers were used: hβ-Actin for 5′ TGACGGGGTCACCCAC AC-3′; hβ-Actin rev 5′-TAAAACGCAGCTCAGTAACAG TCCG-3′; mβ-Actin for 5′-TGGAATCCTGTGGCAT CCATGAAAC-3′; mβ-Actin rev 5′-TAAAACGCAGCT CAGTAACAGTCCG-3′; hSELENBP1 for 5′-ATCTGG CCACTGTGGATGTT-3′; hSELENBP1 rev 5′-CACCAC ATAGATGCGAGAGGA-3′; mSELENBP1 for 5′-GCAC TGAAGCCCCGGATTAT-3′; mSELENBP1 rev 5′-ACATC CACCACGTAGATGCG-3′; hGPX1 for 5′-CGCC AAGAACGAAGAGATTC-3′; hGPX1 rev 5′-AAAGT TCCAGGCAACATCGT-3′; hCDH-1 for 5′-ACCAGGAC TTTGACTTGAGC-3′; hCDH-1 rev 5′-GACTAGCAGC TTCGGAACC-3′; hCDH-2 for5′-TGGATGAAGATGG CATGG-3′; hCDH-2 rev5′-AGGTGGCCACTGTGCTTAC -3′; hVimentin for 5′-TGGCCGACGCCATCAACACC-3′; hVimentin rev 5′-CACCTCGACGCGGGCTTTGT-3′. Each sample was analyzed in duplicate. The target cDNA was normalized to β-actin levels.

### RNA-sequencing

For gene expression analysis RNA samples of two nevi and two melanoma samples from Tg(*Grm1*) mice were analyzed by RNA-sequencing. For this purpose, four poly-A RNA sequence libraries were generated. Single-end reads of 100 bp were sequenced at the Center of Excellence for Fluorescent Bioanalytics (KFB) (Regensburg, Germany; http://www.kfb-regensburg.de) with HiScanSQ (TruSeq SBS kit v3; Illumina, San Diego, CA, USA) technology from Illumina. Sample reads were aligned to the mouse reference genome mm9 (NCBI37) from UCSC (University of California, Santa Cruz, CA, USA) using Rsubread [34] with the default parameters and the in-built annotation data for mm9. The sequence data are available at NCBI, BioProject PRJNA237546. Gene counts based on Entrez genes (http://www.ncbi.nlm.nih.gov/gene) were generated with feature Counts [34] using default parameters for single end data. Differential gene expression was performed using edgeR [35] Genes with a false discovery rate below 0.01 were considered significantly differentially expressed.

### Protein isolation and western blot analysis

Cells and tissues were lysed in 200 μl RIPA buffer (Roche, Mannheim, Germany) for 15 min at 4°C and cell debris was separated via centrifugation at 13,000 rpm and 4°C for 10 min. Protein concentration was determined using the Pierce BCA Protein Assay Kit (Thermo Fisher Scientific, Rockford, USA). For each sample, 40 μg of total lysate were separated on 10% SDS-PAGE gels and subsequently transferred onto a PVDF membrane. After blocking for 1 h with 5% BSA/PBS the membrane was incubated overnight (4°C) with one of the following antibodies: anti-SELENBP1 (Abcam, Cambridge; 1:1000), anti-β-actin (Sigma-Aldrich, Missouri, USA; 1:5000), anti-GXP1 (Thermo Fisher Scientific; 1:1000) or anti-GAPDH (Cell Signaling Technology, Frankfurt a.M.; 1:1000). After washing three times with TBS-T, the membrane was probed with an alkaline phosphate-coupled secondary antibody (anti-rabbit AP or anti-mouse AP, Cell Signaling Technology, Frankfurt a.M., Germany; 1:4000 and 1:3000, respectively) for 1 h. Finally, the membrane was washed three times with TBS-T and the immunoreaction was visualized using NBT/BCIP (Life technologies, Carlsbad, USA).

### Immunohistochemical analysis

Standard 5 μm sections of formalin-fixed and paraffin-embedded tissue blocks were used for immunohistochemistry of murine and human tissue samples. Immunohistochemical staining was performed using anti-SELENBP1 antibody (Abcam, Cambridge, UK; 1:100) and the Envision^TM^ system (Dako, Hamburg, Germany) for human and the Permanent HRP Green Kit (Zytomed, Berlin, Germany) for murine tissue samples. Immunofluorescence staining of cells was performed from each cell line seeded (5 × 10^4^ cells) onto eight-well chamber slides (Corning Incorporated, Corning, USA) and incubated overnight at 37°C. After 15 min fixation with 4% PFA, one washing step with PBS, 5 min, incubation with 0.1% Triton-X-100 followed. Subsequently, cells were washed three times with PBS and blocked for 1 h with 1% BSA/PBS. Incubation with the anti-SELENBP1 antibody (Abcam, Cambridge, UK; 1:120) continued over night at 4°C, and after three washing steps the secondary Alexa Fluor 488 anti-rabbit antibody (Life technologies, Carlsbad, USA; 1:500) was added for 1 h. Finally, cells were washed again with PBS and VECTASHIELD^TM^ Slide Mounting Medium with DAPI (Vector Laboratories Inc., Burlingame, USA) was added for mounting.

### Statistical analysis

Results are shown as the mean ± standard error of the mean or percent, and statistical significance was determined using the Student's unpaired *t*-test (Figures 1, 2, 3). Comparison between more than two groups was made using a one-way ANOVA (Kruskal-Wallis test) analysis of variance (Figure 4 and 5) calculated with GraphPad Prism 7 (GraphPad Software, Inc., San Diego, USA). A *p*-value < 0.05 was considered as statistically significant (ns: not significant, ^*^p < 0.05, ^**^p < 0.01).

## SUPPLEMENTARY MATERIALS FIGURE



## Abbreviations

SELENBP1: Selenium-binding protein 1
GPX1: Glutathione peroxidase 1
NHEM: normal human epidermal melanocytes
NS: normal skin
PT: primary tumor
MET: melanoma metastasis
MSA: methylseleninic acid
