# Supplementary material for: Selenium-binding protein 1 is down-regulated in malignant melanoma
Source: Oncotarget. 2018 Jan 2;9(12):10445–56. doi: 10.18632/oncotarget.23853 (PMC5828193; doi:10.18632/oncotarget.23853)
Supplement: Supplementary file 1 [file oncotarget-09-10445-s001.pdf]

Selenium-binding protein 1 is down-regulated in malignant melanoma

SUPPLEMENTARY MATERIALS

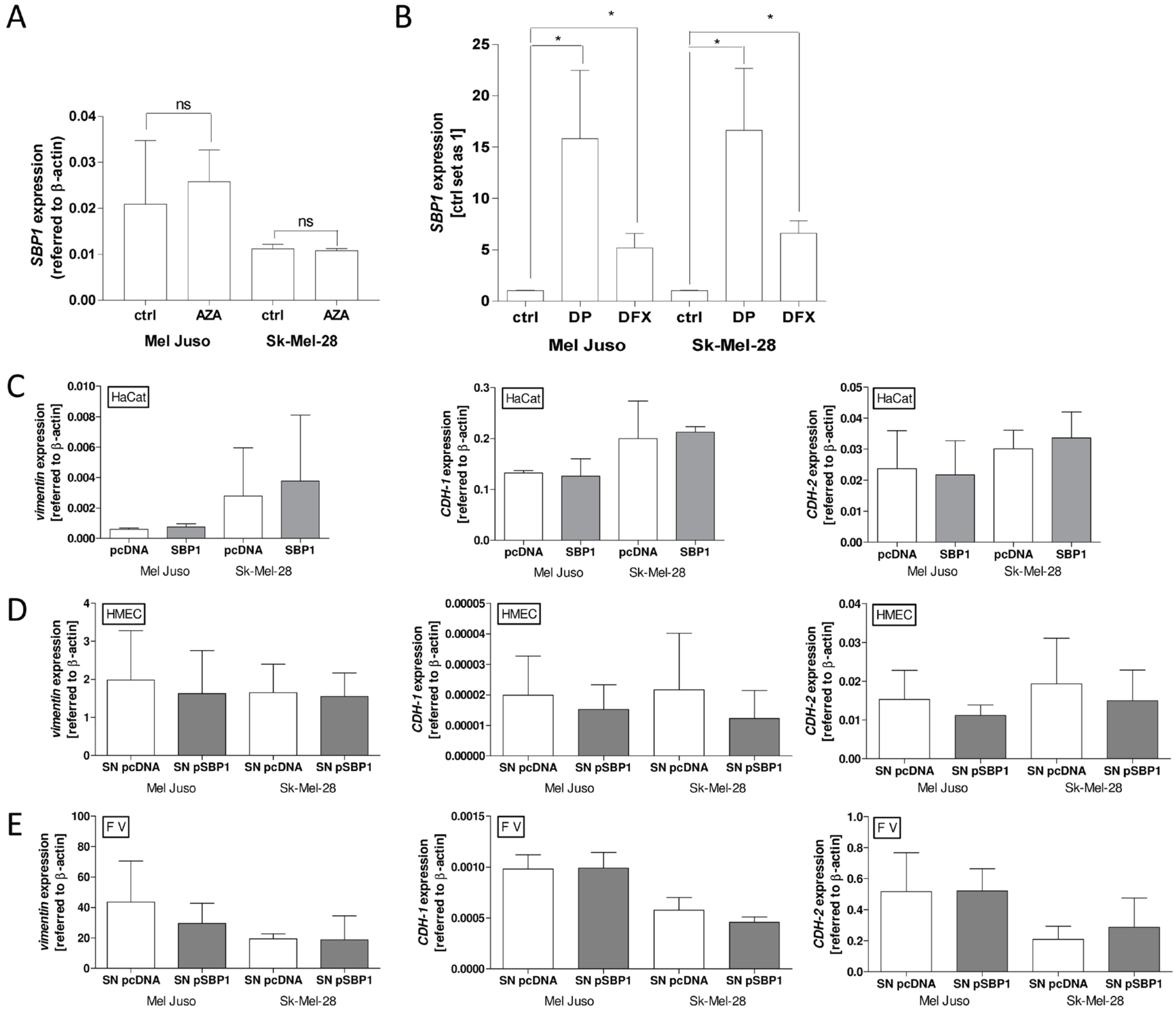

Supplementary Figure 1: (**A**) mRNA analysis of *SELENBP1* expression after 5-Aza-2′-deoxycytidine (AZA) and TSA treatment. (**B**) mRNA analysis of *SELENBP1* expression after induction of hypoxia with Desferrioxamine (DFX) and 2, 2-dipyridyl (DP). (**C**) mRNA analysis of Vimentin, CDH-1 and CDH-2 expression after incubating HaCaT cells with supernatant (SN) of melanoma cells which re-expressed SELENBP1 (pSBP1). (**D**) mRNA analysis of Vimentin, CDH-1 and CDH-2 expression after incubating HMEC cells with supernatant (SN) of melanoma cells which re-expressed SELENBP1 (pSBP1) or a control vector. (**E**) mRNA analysis of Vimentin, CDH-1 and CDH-2 expression after incubating F V cells with supernatant (SN) of melanoma cells which re-expressed SELENBP1 (pSBP1).
